# Single-Cell RNA Sequencing Reveals Cardiac Fibroblast-Specific Transcriptomic Changes in Dilated Cardiomyopathy

**DOI:** 10.3390/cells13090752

**Published:** 2024-04-26

**Authors:** Adam Russell-Hallinan, Oisín Cappa, Lauren Kerrigan, Claire Tonry, Kevin Edgar, Nadezhda Glezeva, Mark Ledwidge, Kenneth McDonald, Patrick Collier, David A. Simpson, Chris J. Watson

**Affiliations:** 1 Wellcome-Wolfson Institute for Experimental Medicine, Queen’s University Belfast, Belfast BT9 7BL, UK; a.russell-hallinan@qub.ac.uk (A.R.-H.); claire.tonry@qub.ac.uk (C.T.); k.edgar@qub.ac.uk (K.E.); david.simpson@qub.ac.uk (D.A.S.); 2 School of Medicine, UCD Conway Institute, University College Dublin, D04 V1W8 Dublin, Ireland; nadezhda.glezeva@ucd.ie (N.G.); kenneth.mcdonald@ucd.ie (K.M.); 3 STOP-HF Unit, St Vincent’s Healthcare Group, D04 T6F4 Dublin, Ireland; mark.ledwidge@ucd.ie; 4 Department of Cardiovascular Medicine, Cleveland Clinic, Cleveland, OH 44195, USA; colliep@ccf.org

**Keywords:** heart failure, dilated cardiomyopathy, cardiac fibroblasts, single-cell RNA sequencing

## Abstract

Dilated cardiomyopathy (DCM) is the most common cause of heart failure, with a complex aetiology involving multiple cell types. We aimed to detect cell-specific transcriptomic alterations in DCM through analysis that leveraged recent advancements in single-cell analytical tools. Single-cell RNA sequencing (scRNA-seq) data from human DCM cardiac tissue were subjected to an updated bioinformatic workflow in which unsupervised clustering was paired with reference label transfer to more comprehensively annotate the dataset. Differential gene expression was detected primarily in the cardiac fibroblast population. Bulk RNA sequencing was performed on an independent cohort of human cardiac tissue and compared with scRNA-seq gene alterations to generate a stratified list of higher-confidence, fibroblast-specific expression candidates for further validation. Concordant gene dysregulation was confirmed in TGFβ-induced fibroblasts. Functional assessment of gene candidates showed that *AEBP1* may play a significant role in fibroblast activation. This unbiased approach enabled improved resolution of cardiac cell-type-specific transcriptomic alterations in DCM.

## 1. Introduction

Cardiomyopathies are a class of diseases characterized by structural and functional abnormalities of the myocardium that cause significant morbidity, mortality, and economic strain on global healthcare systems. They are classified into different types and subtypes based on the underlying pathophysiological changes that result in either hypertrophic, restrictive, or dilated cardiac phenotypes. DCM is one of the most common causes of HF and is characterized by ventricular dilation and depressed myocardial performance in the absence of hypertension, congenital, valvular, or ischemic heart disease [1]. The prevalence of DCM is approximately 1:250–500 in the adult population [2], with a 5-year mortality of up to 20% [3] and a significant increase in new cases recorded over the last decade [1,4].

DCM is a multifactorial disease, influenced by both genetic factors (monogenic or complex) accounting for up to 35–50% [3], and environmental factors (inflammation, infection, toxins, and chemotherapeutic agents) [5]. DCM can present as clinical systolic heart failure with reduced ejection fraction (HFrEF) with arrhythmias that may lead to sudden death [3]. Irrespective of underlying etiology, adverse cardiac remodeling is the hallmark feature of DCM, with defining pathological features such as myocardial inflammation, diffuse fibrosis, compensatory hypertrophy, and chamber enlargement that lead to systolic dysfunction [5,6]. Although there have been considerable improvements in both pharmacological and device-based treatments for DCM to reduce symptoms of HF and improve cardiac function, morbidity and mortality for patients with DCM are still unacceptably high, with one- and five-year mortality rates in DCM at approximately 25–30% and 50%, respectively [5], and 15-year follow-up demonstrating a survival rate of 34% [7].

Aberrant transcriptional and functional changes in multiple cardiac cell types, including cardiomyocytes, fibroblasts, endothelial cells, mural cells, and immune cells, are noted to underlie the pathological phenotype of a DCM heart [6]. More detailed characterization of cell-specific alterations is required to guide the much-needed development of novel diagnostic and therapeutic targets, leading to improved disease management and clinical care of patients with DCM.

ScRNA-seq enables the high-throughput measurement of gene expression in thousands of individual cells simultaneously and its application facilitates the study of maladaptive changes in individual cell types within complex tissues. Use of this technology is increasing exponentially, with thousands of scRNA-seq data sets having been generated in various complex disease settings such as cancer, infection, autoimmune disorders, and more recently HF. The rapid increase in scRNA-seq is matched by a proliferation of single-cell library preparation platforms, sequencing strategies, and computational workflows, the combination of which gives rise to quantitatively and qualitatively diverse datasets. In addition to continued improvements in analytical sensitivity and efficiency in ever-larger datasets, recent computational advances have increased the capacity to harmonize between datasets, regardless of initial scRNA-seq strategy. Coupled with the increase in published single-cell data in repositories, these computational developments raise the prospect of revisiting datasets of interest, both by employing more advanced analytical tools and by leveraging more recent gold-standard reference publications, to enhance the interpretation of published datasets and provide novel insights.

In this study, we aimed to detect cell-type-specific transcriptomic alterations implicated in DCM. Recent advances in single-cell analytical tools were employed to perform an integrated analysis of the publicly available adult heart scRNA-seq datasets. Comparative analysis of heart scRNA-seq datasets to whole-RNA-Seq from an independent cohort of DCM patients enabled the identification of DCM-specific transcriptional changes that occur in disease-relevant cell types. These genetic changes, and their relevance to cardiac functionality, were investigated further in cardiac fibroblasts.

## 2. Materials and Methods

### 2.1. Data Retrieval and Pre-Processing

Single-cell RNA-seq datasets for non-HF (n = 14) and DCM (n = 4) adult human heart samples were retrieved from Gene Expression Omnibus (GEO) under accession codes GSE109816 and GSE121893. Patient demographic information is available in Supplementary Table S1, indicating a mean age of 42.8 and 41.3 years in non-HF and DCM, respectively. The initial dataset of 12,429 cells was subject to additional cell quality filtering, excluding cells with >25% reads mapping to ribosomal genes, >30% reads mapping to a single gene, <750 unique genes, and <2000 total counts, resulting in a filtered dataset of 10,242 cells (6675 control and 3567 HF).

### 2.2. Normalization, Integration, and Clustering

Cells were partitioned according to their individual of origin and processed in Seurat (version 3.2.3 [8]) using the SCTransform package (version 0.3.2 [9]) for normalization and variance stabilization, regressing variance arising from cellular read depth and ribosomal counts, and subsequently in per-individual iterative pairwise integration using the top 3000 variable genes. Principal Component Analysis was performed on the integrated dataset, with the first 50 components used as inputs in UMAP and SNN clustering. Clustering was carried out iteratively from resolutions of 0.5 to 2.0 at increments of 0.25, with clusters at resolution 0.75 most closely matching distinct cellular populations observed in UMAP.

### 2.3. Gene Expression Analysis

Cluster marker genes were detected using the MAST package (version 1.24.1 [10]) using log fold-change and fraction-expressing thresholds of 0.5 and 0.33, respectively, with clusters annotated by cell type accordingly. MAST was used in differential expression analysis within cell types between DCM and non-HF cells, with a log fold-change threshold of 0.15 and a minimum expression fraction of 0.1. Genes passing these thresholds and with a Bonferroni-corrected *p* value < 0.05 were considered significantly differentially expressed between conditions.

### 2.4. Reference Label Transfer

Adult human single-cell data from Heart Cell Atlas [7] was used as a reference dataset in the Seurat Label Transfer to project predicted cell-type annotations onto the query HF dataset based on the gene expression correlation for every cell.

### 2.5. RNA Sequencing of DCM Patient Cardiac Tissue

The study conformed to the principles outlined in the Declaration of Helsinki. Ethical Approval for data collection and use of tissue was obtained from the Cleveland Clinic Institutional Review Board. Cardiac interventricular septal (IVS) tissue was surgically removed from nine patients with DCM that underwent orthotropic cardiac transplantation (OCT). Nine patients represented an age- and gender-matched control group with non-HF hearts who died of non-cardiac causes. These patients donated hearts for OCT. The patient characteristics for these groups have been previously reported [11]. Surgically excised cardiac tissue was immediately snap frozen in liquid nitrogen and stored at −80 °C until required. Approximately 30 mg of tissue was physically homogenized via the use of a motorized pellet pestle (Sigma-Aldrich, Dorset, UK). Total RNA was extracted from IVS tissue homogenate using an RNeasy Fibrous Tissue Mini Kit (Qiagen, Manchester, UK) isolation kit following manufacturers’ instructions. To confirm the presence of RNAs, samples were quantified using the Qubit™ RNA Assay Kit (Thermo Fisher, Leicestershire, UK).

Library preparation and next-generation sequencing were carried out by the Queen’s University Genomics Core Technology Unit. Libraries were prepared from 1 µg of RNA using TruSeq^®^ Stranded mRNA Library Prep kit (Illumina, Cambridge, UK) following the manufacturers’ instructions. Library concentrations were measured using a Qubit™ dsDNA HS Assay Kit (Thermo Fisher, Leicestershire, UK). The quality and concentration of libraries were determined using a Fragment Analyzer (Advanced Analytical, Cheshire, UK). Libraries were sequenced on a NextSeq 500 System (Illumina, Cambridge, UK).

An analytical workflow on CLC Bio Genomics workbench v10.1.1 (QIAGEN) was used for sequence analyses. Imported raw sequence FASTQ files were assessed for QC, trimmed, and extracted; reads were then mapped to HG19. Mature annotated sequences were assigned experimental groups (non-HF vs. DCM). Gene mapping matrices were normalized to RPMM and compared between experimental groups for differential gene expression. Proportion-based statistical analysis (Baggerly’s test, Bonferroni corrected) was also carried out on the CLC Bio Genomics workbench package.

### 2.6. Analysis of Mass Spectrometry of Human Left Ventricular Tissue

Proteomic data generated from mass spectrometry-based analysis of left ventricular heart tissue from patients with no heart failure (n = 7) and patients with dilated cardiomyopathy (n = 5) was accessed from ProteomeXchange to evaluate the protein-level expression changes of AEBP1 in association with DCM (PXD008394) [12].

### 2.7. Cell Culture

Primary human ventricular cardiac fibroblast (HCF) cells were purchased from ScienCell Research Laboratories; primary cells isolated from two individual donors were included in this study. Cells were cultured and maintained in Dulbecco modified Eagle medium (DMEM; Gibco, Leicestershire, UK), supplemented with 10% fetal bovine serum (Gibco, Leicestershire, UK) and penicillin–streptomycin antibiotics (Gibco) in a 5% CO_2_ humidified incubator at 37 °C. Cells were not expanded beyond 15 population doublings in line with suggested conditions provided by ScienCell Research Laboratories. When required, HCF cells were treated for 72 h with either vehicle or 5 ng/mL human recombinant transforming growth factor β1 (TGF-β1; Promokine, Heidelberg, Germany) in serum free media. HCF cells were also stimulated with TGF-β1 in the presence of either 25 nM siRNA (siRNA) or 25 nM scrambled non-targeting control (scr), for genes that were identified as upregulated in DCM (*LTBP2* and *AEBP1*).

### 2.8. Quantitative Real-Time Polymerase Chain Reaction (RT-qPCR)

Total RNA was extracted from cells using the High Pure RNA Extraction kit (Roche, Sussex, UK) according to the manufacturers’ instructions. Subsequently, 1 µg DNase-free RNA was reverse transcribed using the High Capacity cDNA Reverse Transcription Kit (Thermo Fisher Scientific).

Quantitative RT-PCR (RT-qPCR) was carried out using fluorescent SYBR Green (Roche, Sussex, UK). Primers were designed using Primer Blast (exon and intron spanning when possible) or purchased from Sigma and are shown in Supplementary Table S2. Ct values were measured using a LightCycler 480 sequence detector (Roche, Sussex, UK). Relative fold change in gene expression was calculated with the ΔΔCT method. Expression of *B2M*, *HPRT*, and *GAPDH* were used as endogenous controls for normalization.

### 2.9. Western Blotting

Protein expression levels were measured using Western blotting. HCF cell lysates were lysed in RIPA lysis buffer. Protein concentrations were measured using BCA protein Assay (Promega, Southampton, UK). Protein lysates (25 µg) and cell supernatants (20 µL) were electrophoresed on 10% sodium dodecyl sulfate-polyacrylamide gels, and then transferred onto polyvinylidene fluoride (PVDF) membranes (BioRad, Hercules, CA, USA) using a gel transfer device. PVDF membranes were blocked in 5% non-fat milk/tris-buffered saline with 0.01% Tween™ 20 (TBS-T) for 1 h and then incubated with primary antibodies against α-smooth muscle actin (Merck, Dorset, UK, 1:5000) overnight at 4 °C. After washing six times with TBS-T, the membrane was stained with anti-mouse IgG (CellSignalling, Beverly, US, 1:10,000) for 1 h. Blots were washed (as described above) and developed using SuperSignal™ West Pico PLUS Chemiluminescent Substrate (ThermoScientific, Singapore) and visualized using the Chemgene Film Processor (Protec, Oberstenfeld, Germany). Protein expression levels were normalized to GAPDH (ProteinTech, Manchester, UK 1:10,000).

### 2.10. Statistical Analysis

All statistical analyses were performed using R (3.6.3) and Graphpad Prism software (Version 6). For in vitro investigation, normality was assessed using the Kolmogorov–Smirnov test, with Gaussian distributed data represented as the mean and standard deviation of the mean (SD), while non-normally distributed data are presented as the median and interquartile range (IQR). A two-sided, unpaired Student’s *t*-test was used for two-group comparison (normal distribution). For non-normally distributed data, a Mann–Whitney U test was used. Values of *p* < 0.05 and FDR < 0.05 were accepted as statistically significant.

## 3. Results

### 3.1. Optimization and Recharacterization of Single-Cell Transcriptomic Changes in DCM

**Figure 1:**
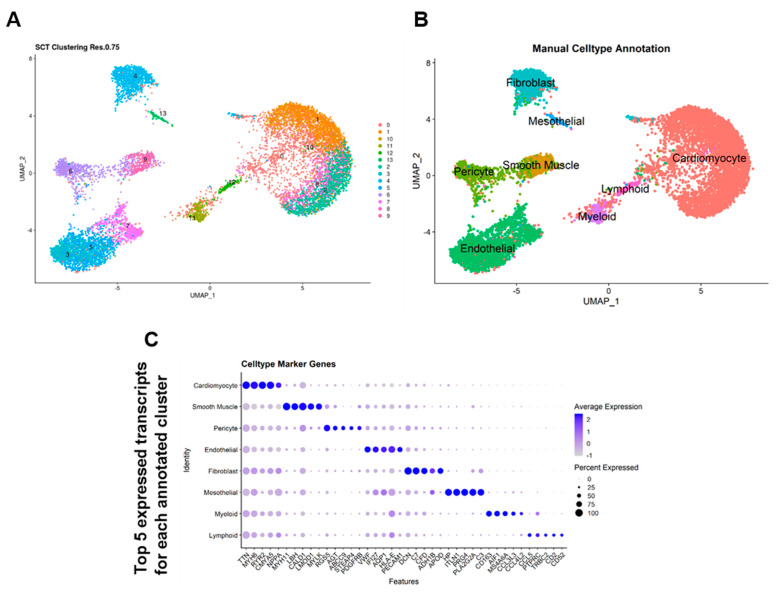
Enhanced workflow and unbiased automatic cell labelling of human cardiac scRNA data enhances identification of cardiac cell populations. UMAP reduction of full Wang et al.’s dataset labelled with (**A**) unsupervised SNN clustering at 0.75 resolution and (**B**) after semi-unsupervised cell-type annotation. (**C**) Top 5 most highly expressed transcripts for each annotated cell cluster.

Unsupervised clustering analysis identified eight clearly distinct cell populations (Figure 1A,B). Using the non-HF cohort alone, the eight cell populations were manually annotated using literature-derived annotation using the most enriched marker genes in each population: cardiomyocytes (*TTN*, *MHY6*, *RYR2*, *NPPA*), fibroblasts (*DCN*, *C7*, *CFD*, *ADH1B*, *APOD*), endothelial cells (*VWF*, *IFI27*, *AQP1*, *HLA-E*, *PECAM1*), smooth muscle cells (*MYH11*, *LBH*, *CALD1*, *LMOD1*, *MYLK*), pericytes (*RGS5*, *AGT*, *ABCC9*, *STEAP4*, *PDFGRB*), mesothelial cells (*HP*, *ITLN1*, *PRG4*, *PLA2G2A*, *C3*), myeloid cells (*CD163*, *AIF1*, *MS4A6A*, *CCL3L3*, *CCL4L2*), and lymphoid cells (*CCL5*, *PTPRC*, *TRBC2*, *CD2*, *CD52*) (Figure 1C). A comprehensive set of markers for unsupervised clusters and annotated cell types can be found in Supplementary Tables S3 and S4. Automated cell-type annotation with Seurat label transfer using the Heart Cell Atlas project6 as a reference identified the same eight cellular populations (Supplementary Figure S1).

As a neuronal cluster was reported in Heart Cell Atlas, supervised examination for neuronal marker genes was applied (*NRXN1*, *NRXN3*, *KCNMB4*, Supplementary Figure S2). Expression of these markers was low, occurring in few cells across various clusters, indicating the absence of a specific neuronal cluster in this dataset.

### 3.2. Cellular Transcriptional Changes in DCM

**Figure 2:**
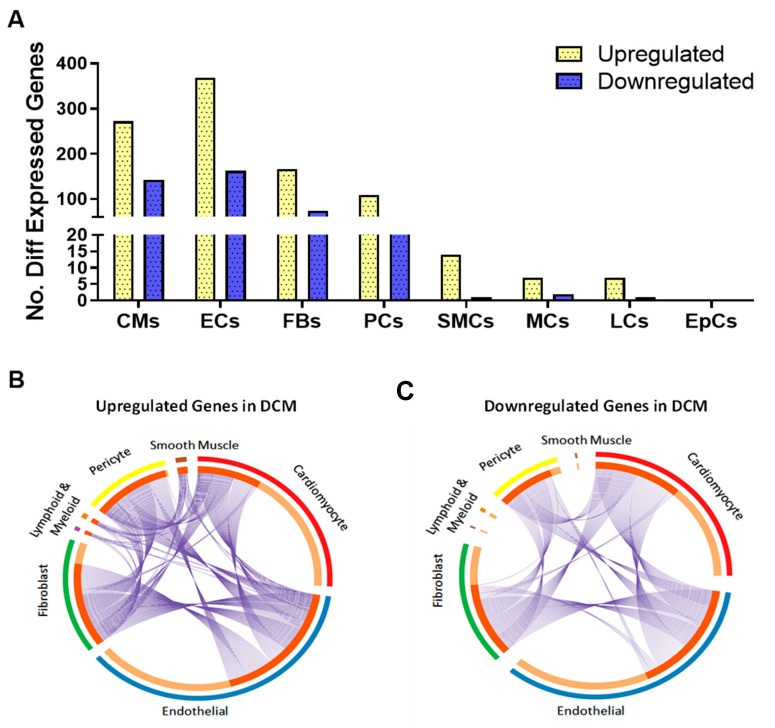
Differential transcriptional changes in cardiac cell types in DCM. (**A**) Number of differentially expressed transcripts in each cardiac cell population in DCM. Circos plots of differentially expressed genes that were (**B**) upregulated and (**C**) downregulated in each of the cardiac cell populations. Blue lines indicate transcripts that are conserved between different cell populations.

Identification of transcriptional differences between the non-HF cohort and DCM cohort was carried out for each cardiac cell type. Major differences in expression in DCM were identified in four of the predominant cell types: cardiomyocytes (272 upregulated, 143 downregulated genes), endothelial cells (369 upregulated, 162 downregulated), fibroblasts (166 upregulated, 74 downregulated), and pericytes (109 upregulated, 41 downregulated). We identified a small number of genes differentially expressed in DCM in the other cell populations, including smooth muscle cells (14 upregulated, 1 downregulated), myeloid cells (7 upregulated, 2 downregulated), and lymphoid cells (7 upregulated, 1 downregulated) (Figure 2A). The mesothelial cell population did not exhibit any differential gene expression reaching significance between non-HF and DCM, with this outcome likely arising from the low numbers of mesothelial cells and variation in their proportions between individual heart samples.

Selected genes commonly altered in cardiomyopathies were queried for differential expression (Supplementary Figure S3), indicating in particular increased expression of natriuretic peptides, myosin heavy chain, and troponin genes, and a decrease in myosin light chain genes across the major cell types. The expression of 11 genes reported to have strong genetic links to DCM [13] was also queried, of which 7 (*FLNC*, *DES*, *MYH7*, *PLN*, *TTN*, *TNNC1,* and *TNNT2*) demonstrated significant dysregulation in DCM (Supplementary Figure S4).

The full list of differentially expressed genes identified in DCM for each cardiac cell type is found in Supplementary Table S5. Several DEGs showed overlap across two or more cardiac cell types in DCM (Figure 2B). For example, *HES1* expression was significantly upregulated in endothelial cells, fibroblasts, and pericytes, and MYL3 expression was significantly downregulated in cardiomyocytes, fibroblasts, endothelial cells, and pericytes. However, we identified subsets of DEGs that were uniquely dysregulated for each cardiac cell type in DCM (Figure 2B).

### 3.3. Identification and Validation of Fibroblast-Specific DCM Transcriptomic Changes

**Figure 3:**
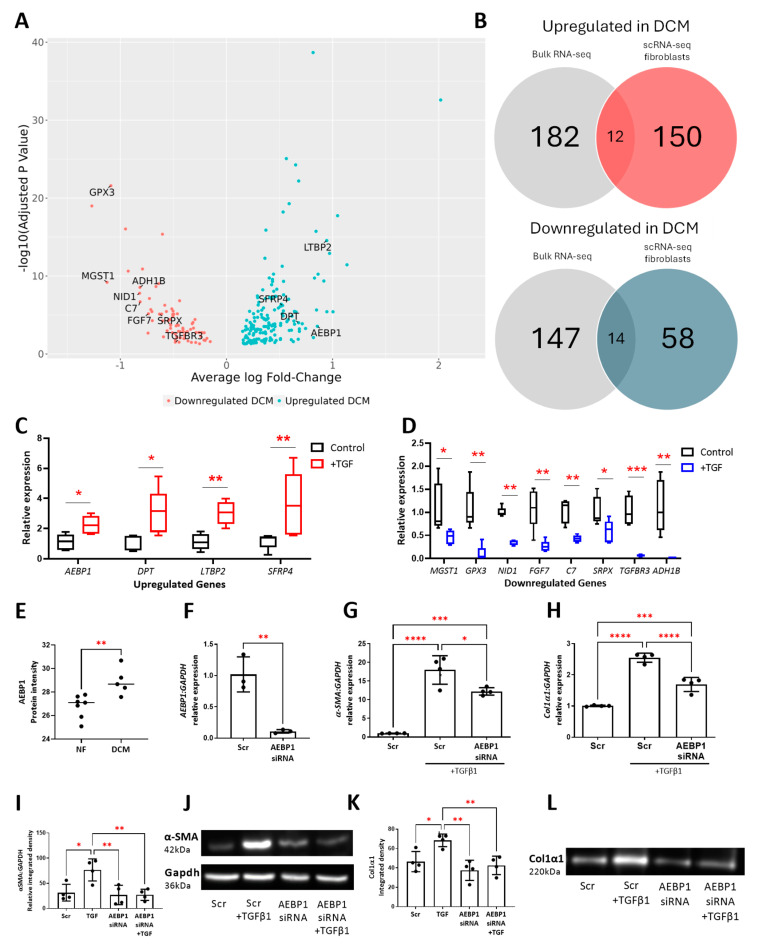
Identification and in vitro validation of fibroblast-specific differentially expressed genes in DCM. (**A**) Volcano plot representing differentially expressed genes in cardiac fibroblasts from DCM hearts. (**B**) Venn diagram depicting identification of disease-relevant candidate genes by comparing DEGs from bulk RNA-seq of heart tissue with scRNA-seq fibroblast cell data. In vitro validation of disease-relevant candidates that show significant (**C**) upregulation and (**D**) downregulation in response to 72 h TGFβ activation (n = 5). (**E**) Proteomic data generated from mass spectrometry-based analysis of LV heart tissue from non-failure patients (NF) (n = 7) and dilated cardiomyopathy patients (DCM) (n = 5). (**F**) RT-PCR analysis of *AEBP1* in HCFs transfected with 25 nM *AEBP1* siRNA (or scrambled control) (n = 3). RT-PCR analysis of (**G**) αSMA and (**H**) *col1α1* in HCFs transfected with 25 nM *AEBP1* siRNA (or scrambled control) in the presence or absence of TGFβ1 (n = 4). Protein levels of (**I**) αSMA and (**K**) col1α1 measured in lysates (n = 4) and supernatants (n = 4), respectively, from HCFs transfected with 25nM *AEBP1* siRNA (or scrambled control) in the presence or absence of TGFβ1 using Western blot and densitometry analysis. (**J**,**L**) show representative images of Western blots. * indicates *p* =< 0.05, ** indicates *p* =< 0.005, *** indicates *p* =< 0.0005, **** indicates *p* =< 0.00005).

Fibroblasts are one of the leading cell types involved in pathological cardiac remodeling in the DCM heart. ScRNA-seq analysis of fibroblasts revealed unique transcriptional changes in DCM with 166 upregulated and 74 downregulated DEGs identified (Figure 3A). Pathway analysis revealed that genes upregulated in DCM are associated with TGFβ receptor signaling pathway (GO:0007179), extracellular matrix organization and remodeling (GO:0030198, GO:0030199), regulation of fibroblast proliferation (GO:0048146), response to oxidative stress (GO:0006979, GO:0000302) and cytokine-mediated signaling (GO:0019221) (Supplementary Table S6). Transcripts which were downregulated in fibroblasts from DCM hearts were associated with cardiac tissue morphogenesis (GO:0055008, GO:0055010), mesenchymal cell proliferation (GO:0010463), migration in cardiac development (GO:0060973), mitochondrial electron transport (GO:0006122, GO:0006123), and response to lipid hydroperoxide (GO:0006982) pathways. Pathway analysis of differentially expressed transcripts was also conducted on other prominent cell types, including cardiomyocytes, endothelial cells, and pericytes that were analyzed from this scRNA-seq dataset (Supplementary Table S6).

To independently identify DCM-related transcriptional changes in fibroblasts, cardiac tissue was profiled from a separate cohort of nine DCM patients and nine non-HF patients using RNA-seq [10]. Principal component analysis revealed a clear separation in transcriptional profiles between the DCM and non-HF cohort; there were 182 upregulated genes and 147 downregulated genes in dCM compared to NF cardiac tissue (Supplementary Figure S5). Intersecting these differentially expressed myocardial DCM candidate genes (Supplementary Table S7) with those identified in fibroblasts from the single-cell analysis revealed n = 12 upregulated and n = 14 downregulated gene candidates which showed the same directional change in expression (Figure 3B). To further examine these fibroblast-specific transcriptional changes in DCM, their expression using RT-qPCR was measured in an in vitro model of TGFβ-induced activation, a cytokine known to be present at high levels in DCM myocardium [14] and which was found to be upregulated in NF vs DCM bulk RNAseq (Supplementary Table S7). Activation of cardiac fibroblasts in vitro with TGFβ was confirmed by induction of profibrotic markers, including alpha smooth muscle actin (*αSMA*) and collagen 1 alpha 1 (*col1α1*). Ten candidate genes demonstrated the same change in expression upon TGFβ treatment as detected in DCM. Four genes; *AEBP1*, *LTBP2*, *SFRP4*, and *DPT* showed a significant elevation in expression in response to TGFβ (Figure 3C) and expression of *TGFΒR3*, *ADH1B*, *GPX3*, *MGST1*, *NID1*, and *C7* was significantly reduced (Figure 3D). The remaining candidate genes examined in vitro showed opposing or no change in mRNA expression. We also evaluated changes in candidate protein-level expression in proteomic data generated from mass spectrometry-based analysis of LV heart tissue from patients with no heart failure (n = 7) and patients with dilated cardiomyopathy (n = 5) [12] and found that only AEBP1 was upregulated in DCM at the protein level (NF vs. DCM, *p =* 0.0062 **), concordant with our gene expression data (Figure 3E). The other candidate proteins evaluated in this data set were not significantly different between NF and DCM patients.

To further assess the functional impact of aberrant transcriptional expression on fibroblast activation, we conducted siRNA knockdown of the two highest, differentially expressed candidates (in RNA-seq and scRNA-seq), *AEBP1* and *LTBP2*, and examined their impact on fibrotic factors in HCFs. Transfection of AEBP1 siRNA resulted in a significant 10-fold reduction in *AEBP1* expression (scr vs. siAEBP1, *p =* 0.005 **) (Figure 3F). In response to TGFβ activation, siRNA-targeted knockdown of *AEBP1* resulted in a significant reduction in the expression of *αSMA* (scr vs. siAEBP1, *p =* 0.0154 *) (Figure 3G) and *col1α1* (scr vs. siAEBP1, *p* ≤ 0.0001 ***) (Figure 3H). The effect of siRNA-targeted knockdown of AEBP1 on the fibrotic response of HCFs at the protein level was assessed using Western blot analysis. Results showed a significant reduction in TGFβ-induced αSMA protein from cell lysates relative to Gapdh protein expression (Figure 3I,J) and reduced col1α1 secretion from HCFs into the cell supernatant (Figure 3K,L). Reduced expression of LTBP2 by siRNA-targeted knockdown demonstrated no functional impact on pro-fibrotic gene activation in response TGFβ activation (Supplementary Figure S6).

## 4. Discussion

Transcriptional profiling of the cellular constituents of the heart under pathological conditions using single-cell genomics offers valuable insights into the molecular changes that underly complex cardiomyopathies, along with the potential to deliver novel diagnostic and therapeutic targets to improve the clinical outcome. In this study, we conducted an integrated analysis of publicly available adult heart scRNA-seq datasets coupled with whole RNA-seq of cardiac tissue to identify and independently validate disease-associated transcriptomic changes in cardiac fibroblasts in DCM.

Previous analysis of the single-cell HF dataset conducted using the Wang et al. system involved high-resolution clustering of cardiac cell populations followed by detection of changes in sub-cluster proportions [15]. Our methodology followed a more conventional approach, with unsupervised clustering at a resolution more closely matching the cellular heterogeneity detected in UMAP, followed by cluster marker gene detection. Clusters were then manually annotated into distinct cardiac cell types based on their expression of canonical marker genes. Combined with refined cell-quality filtering and the use of the intensive batch-correction software SCTransform to more effectively minimize confounding technical effects, this approach resulted in the demonstrably greater resolution of cardiac cellular populations.

Furthermore, our analysis employed reference mapping, benefitting from the recent publication of the human Heart Cell Atlas [6], unavailable at the time of the Wang et al. publication [13], from which curated cell-type annotations could be projected without bias. The strong overlap in cardiac cell types between automated and manual annotations indicates that our updated characterization is robust. More effective clustering enhanced the detection of differential expression and improved cell-type annotation, meaning changes could be attributed to known cardiac cell types with greater specificity, particularly regarding the clear distinction between smooth muscle and pericytes, and between myeloid and lymphoid populations, the separation between which was not described in the original publication. Thus, these analytical changes provided a sounder basis for cell-type-specific differential expression comparison in DCM.

DCM-relevant effects were detected through differential expression analysis (using the scRNA-seq optimized DGE package, MAST) to identify transcriptomic changes more confidently to known cardiac cell types. This contrasts with the approach taken originally by Wang et al., which focused on the detection of putative subclusters for each cell type, and their proportional changes during HF [13]. As their subclusters are not related to known cardiac cell types, the value of changes in cell-type proportion is limited by the difficulty in relating them to DCM pathophysiology. Our workflow’s cell-type-level focus is further advantageous as the greater number of cells in each comparison results in increased power to detect differentially expressed genes in DCM. We, therefore, consider that the cell-type-specific differential expression detected with our updated workflow more reliably reflects disease-relevant changes occurring in DCM.

Fibroblasts are a key cellular contributor to cardiac dysfunction in DCM through pathological myocardial remodeling. Our cell clustering and integration of transcriptomic data at the single-cell and the whole-heart level identified 26 genes that were differentially expressed in fibroblasts in DCM. Of these, 10 genes demonstrated concordant expression with TGFβ activation in vitro. Four genes, *AEBP1*, *LTBP2*, *DPT,* and *SRP4,* were significantly upregulated along with six genes, *TGFBR3*, *NID1*, *C7*, *ADH1B*, *GPX3*, and *MGST1*, that significantly reduced in fibroblasts under pathological conditions.

We then aimed to assess the functional role of genes upregulated in DCM fibroblasts. For two of the four candidates, DPT and SRP4, even in stimulated cells, expression levels were relatively low. We, therefore, theorized that the knockdown of these genes would be unlikely to have a functional effect. In contrast, *AEBP1* and *LTBP2* were the top two highest expressed genes in both the combined RNA-seq and in the scRNA-seq analysis. For this reason, only the functionality of *AEBP1* and *LTBP2* were examined. Transfection of fibroblasts with siRNA significantly decreased the gene expression of both genes. Reduced expression of *LTBP2* did not have an effective impact on fibroblast stimulation by TGFβ. However, *AEPB1* knockdown significantly diminished the TGFβ activation of fibroblasts, evident in a substantially dampened induction of the profibrotic genes *αSMA* and *col1α1* in stimulated cells. These data suggest that *AEBP1* could be an important key player in the development of cardiac fibrosis characteristic of DCM, and could pose as a potential therapeutic target in this condition.

AEBP1 is known to function as a transcriptional repressor and has recently been identified through scRNA-seq analysis as a crucial fibrosis regulator in αSMA-positive myofibroblasts [16]. Combined comparative analysis of GEO (transcriptomic) and PRIDE (proteomic) repository data identified *AEBP1* as differentially expressed at both the gene and protein level as part of a 10-molecule signature of DCM [17]. Data presented in this study support these findings in that AEBP1 was upregulated at both the gene level and the protein level in human cardiac tissue. This signature was associated with biological pathways such as ECM organization, cellular response to stress and response to TGFβ. An in-depth single-nuclei sequencing analysis evaluating lineage-specific changes in pathological cardiac remodeling of hypertrophic cardiomyopathy (HCM) revealed *AEBP1* to be a key gene involved in cardiac fibroblast activation and HCM-associated cardiac fibrosis [18]. Although the results of this study agree with our findings linking *AEBP1* to cardiac fibroblast regulation, their experimental assessment of the functional role of *AEBP1* in cardiac fibroblasts differs from what we observed. They found that *AEBP1* knockdown increased the fibroblast expression of profibrotic factors, and that overexpression of *AEBP1* weakened the TGFβ activation of fibroblasts [16]. This conflicts with our data showing that *AEBP1* knockdown attenuated the TGFβ response, both at the gene expression level and the protein level. This study did not look at the effect of *AEBP1* siRNA knockdown on TGFβ fibroblast activation. Further contradictory evidence on the role of *AEBP1* in the fibrotic response is presented in a study focusing on renal fibrosis. Knockdown of *AEBP1* expression again resulted in a diminished fibrotic response both in cultured renal fibroblasts and in an in vivo model of renal fibrosis, which was then linked to the modulation of the β-catenin signaling pathway [19]. The biological pathways that modulate *AEBP1* expression need to be further elucidated to achieve a fuller understanding of its role in the fibrotic response, specifically in cardiac fibroblast regulation.

LTBP2 is a multifunctional ECM protein with evidence indicating it to have a role in DCM pathogenesis. Recent efforts utilizing scRNA-seq to profile the DCM heart in explanted hearts of paediatric DCM patients revealed an increase in the cardiac fibroblast number and a divergent gene expression profile associated with increasing age [20]. Like our findings, their work demonstrated that fibroblasts from the myocardium of adolescent DCM patients expressed higher levels of *LTBP2* [20]. Further supporting evidence for the role of LTBP2 in promoting a dilated phenotype was recently highlighted by Pang et al. in a doxorubicin-induced rat model of DCM, wherein myocardial expression was significantly upregulated. Knockdown of *LTBP2* using siRNA resulted in reduced myocardial fibrosis, inflammation and oxidative stress through a reduction in NF-kB signaling [21]. Evidence presented in this paper conflicts with these findings in that knockdown of *LTBP2* by siRNA did not impact on the TGFβ induction of cardiac fibroblasts.

Although this study did not examine the functional impact of the remaining candidate genes in cardiac fibroblasts, interestingly, several of the genes are implicated in disease-relevant cellular pathways in other fibrotic pathologies, with limited data in the literature highlighting their direct involvement in DCM pathogenesis. SFRP4 acts as a soluble modulator of Wnt signaling with elevated expression previously demonstrated in the hearts of patients with DCM and coronary artery disease [22]. Increased expression of *SFRP4* in cardiac fibroblasts may in fact act as a protective cellular mechanism in the injured heart as a previous report in the context of ischaemic cardiac injury found that administration of *SFRP4* reduced scar size along with the attenuation of an infarct-related decline in cardiac function [23].

DPT is an ECM protein with functions in cell-matrix interactions and matrix assembly. It is thought to play an important role in wound healing through the induction of fibroblast adhesion and migration through integrin binding [23,24].

TGFBR3 is a transmembrane proteoglycan that is involved in various cellular processes (e.g., proliferation and apoptosis) mediated through binding to TGF-β [25]. Mounting evidence suggests that TGFBR3 may in fact mediate anti-fibrotic activity in fibroblasts through antagonizing the formation of the TGF-β1/TGF-β2 receptor complex along with inhibition of TGF-β1 signaling [26]. The reduction in *TGFBR3* in fibroblasts could be due to repressive actions of non-coding RNAs such as miR-23b-3p and miR-27b-3p26 to amplify the pro-fibrotic myocardial environment in DCM [27]. Therapeutic upregulation of *TGFBR3* in cardiac fibroblasts by simvastatin has been reported to elicit beneficial anti-fibrotic action on fibroblasts and improved cardiac function after myocardial infarction [28]. The application of simvastatin as a potential treatment of DCM is currently being investigated in a phase II clinical study (NCT03775070).

C7 is a serum glycoprotein that forms a membrane attack complex together with complement components C5b, C6, C8, and C9 (C5b-9), performing an important role in innate immunity. Although C7 has not been directly linked to DCM pathology, increased gene expression of the *C5b-9* complex has been found in the myocardium of DCM patients [29] and has been associated with peritubular myofibroblast accumulation in adriamycin nephropathy [30].

ADH1B is involved in the metabolism of a wide variety of substrates, including ethanol, retinol, other aliphatic alcohols, hydroxysteroids, and lipid peroxidation products. A large meta-analysis of 56 epidemiological studies identified the rs1229984 variant of *ADH1B* to carry a more favorable cardiovascular profile associated with a reduced risk of coronary heart disease [31].

NID1 (nidogen) is a basement membrane glycoprotein that interacts with and supports several other components of basement membrane structure, including collagens and laminin proteins [32]. Complete nidogen deficiency in mice leads to premature death associated with cardiac dilation [33]. Furthermore, administration of nidogen and hylauronic acid resulted in improved cardiac function through effects on scar size, scar quality, and improved angiogenesis after ischaemia reperfusion injury [34], suggesting that *NID1* expression in cardiac fibroblasts may facilitate the dilative remodeling of the heart.

Both *GPX3* and *MGST1* genes are known to play an important role in cellular protection from oxidative stress mediated by the antioxidative capacity of glutathione. GPX3 is a selenocysteine-containing enzyme that is primarily expressed in the kidney and functions to rapidly catalyze the reduction of hydroperoxides (e.g., hydrogen peroxide) and lipid peroxidases [35]. Reduced circulating levels of GPX3 was significantly associated with an increased risk of cardiovascular events in patients with atrial fibrillation [36]. Moreover, knockout of *Gpx3* resulted in significant impairment of ventricular function and cardiac fibrosis in a murine model of chronic kidney disease [37]. Mechanistically, activation of cardiac fibroblasts by TGFβ may deregulate the antioxidative capacity in the heart through GPX3 reduction by its action at the TGFR1 receptor rather than downstream Smad 2/3 signaling [38]. Like GPX3, MGST1 is a membrane-bound enzyme that catalyzes the breakdown of a wide range of endogenous and xenobiotic lipophilic electrophiles and peroxides to protect cells from oxidative damage [39]. A recent single-cell-analysis study has identified *MGST1* as a common marker for fibroblasts in multiple organs including heart, colon, bladder, and skeletal muscle [40]. Circulating mRNA expression of expression of *MGST1* was significantly increased 7 days after implantation of a left ventricular assisted device in patients with stage D HF suggesting a potential protective response mechanism to oxidative stress [41]. Our analysis could suggest that suppression of *GPX3* and *MGST1* expression and their antioxidant capacity in cardiac fibroblasts, mediated by TGFβ activation, may facilitate increased oxidative stress, enhancing detrimental remodeling of the myocardium in DCM.

## 5. Conclusions

In conclusion, DCM is a complex pathology involving interactions between multiple cardiac cell populations. Our analysis workflow improved the resolution of cell types at the single-cell level, providing more accurate recapitulation of in vivo tissue heterogeneity. This approach, coupled with transcriptomic data from an independent cohort of DCM cardiac tissue and an in vitro model enabled the robust detection, identification, and validation of unique transcriptomic alterations in cardiac fibroblasts in DCM. Furthermore, our study highlighted *AEBP1* as a gene regulating the pro-fibrotic response of cardiac fibroblasts to TGFβ. Building on other published single-cell studies, identification of these 10 validated genes, showing dysregulation in cardiac fibroblasts, indicates that they may be relevant to DCM pathophysiology and highlights their potential value for further investigation. This may facilitate the development of novel fibroblast-specific targets in advancing therapeutic treatment of DCM.

## Data Availability

The data that support the findings of this study are available from the corresponding author upon reasonable request.

## Supplementary Materials

The following supporting information can be downloaded at: https://www.mdpi.com/article/10.3390/cells13090752/s1, Table S1: Patient demographics for single-cell RNA-seq datasets for non-HF (n = 14) and DCM (n = 4) adult human heart samples, retrieved from Gene Expression Omnibus (GEO) under accession codes GSE109816 and GSE121893. Table S2: Primer sequences used for RT-qPCR validation. Table S3: Unsupervised clustering markers. Table S4: Cell type markers. Table S5: Single-cell RNA-sequencing differential gene expression lists. Table S6: Pathway enrichment analysis. Table S7: Whole tissue RNA-sequencing DCM vs Non-HF differentially expressed gene lists. Figure S1: UMAP reduction of Wang et al. dataset using updated workflow. Figure S2: Supervised examination of neural cell markers in the cardiac single cell dataset. Figure S3: Expression of selected common HF-dysregulated genes across all cardiac cell populations, split into DCM and control (non-HF) cohorts, indicating differences in cell type and cohort expression. Figure S4: Genes with clear genetic association with DCM as reported by Jordan et al. 2021. Figure S5: Differential gene expression in left ventricular cardiac tissue from non-failure versus dilated cardiomyopathy patients. Figure S6: LTBP2 knockdown did not inhibit the TGF-β induced pro-fibrotic response in cardiac fibroblasts.
