# Supplementary material for: Single-Cell RNA Sequencing Reveals Cardiac Fibroblast-Specific Transcriptomic Changes in Dilated Cardiomyopathy
Source: Cells. 2024 Apr 26;13(9):752. doi: 10.3390/cells13090752 (PMC11083662; doi:10.3390/cells13090752)
Supplement: Supplementary file 1 [file cells-13-00752-s001.zip › cells-2897504 supplementary materials resub.pdf]

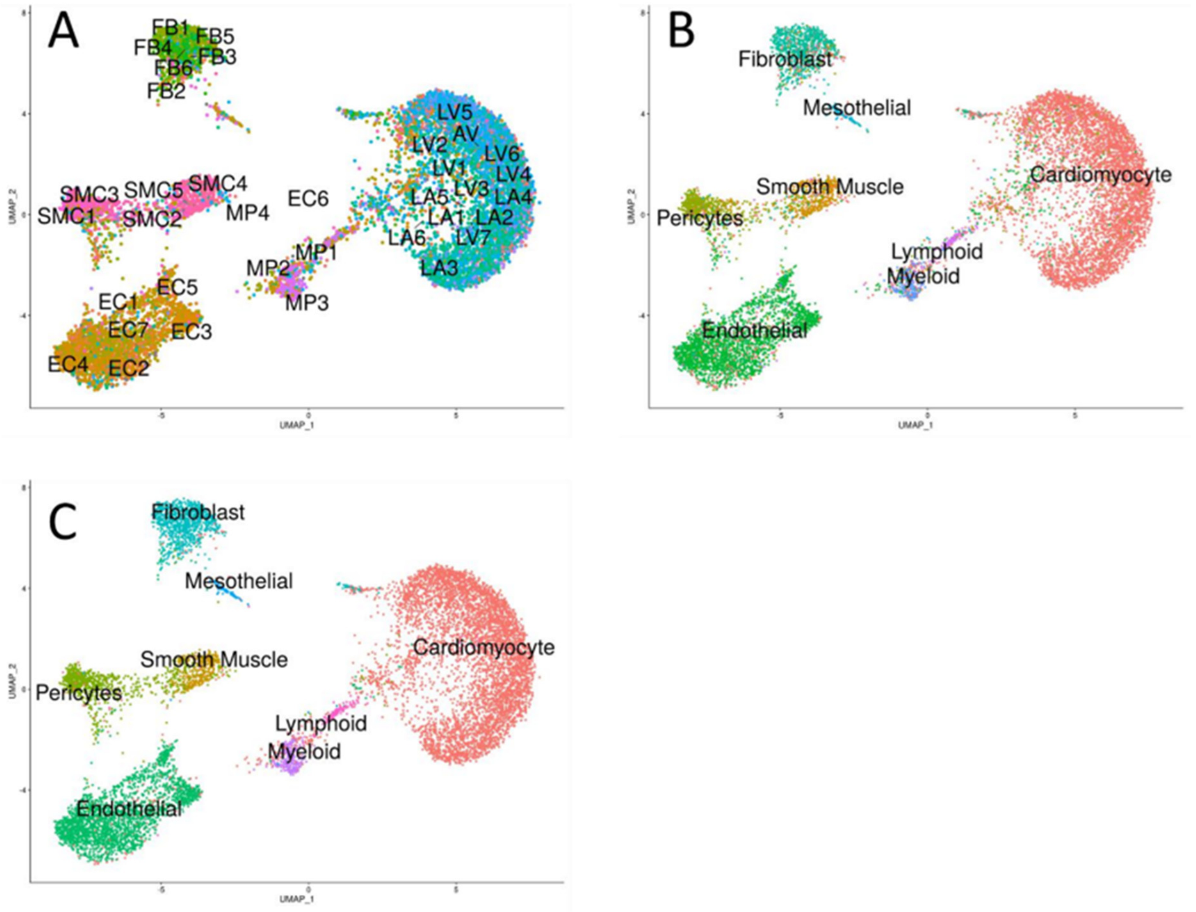

SupplementaryFigureS1: UMAP reduction of Wang et al dataset using updated workflow, cells labelled by A) cell populations including subclusters reported by Wang et al, B) Heart Cell Atlas annotations using Seurat Label Transfer, C) semi-unsupervised cell-type annotation.

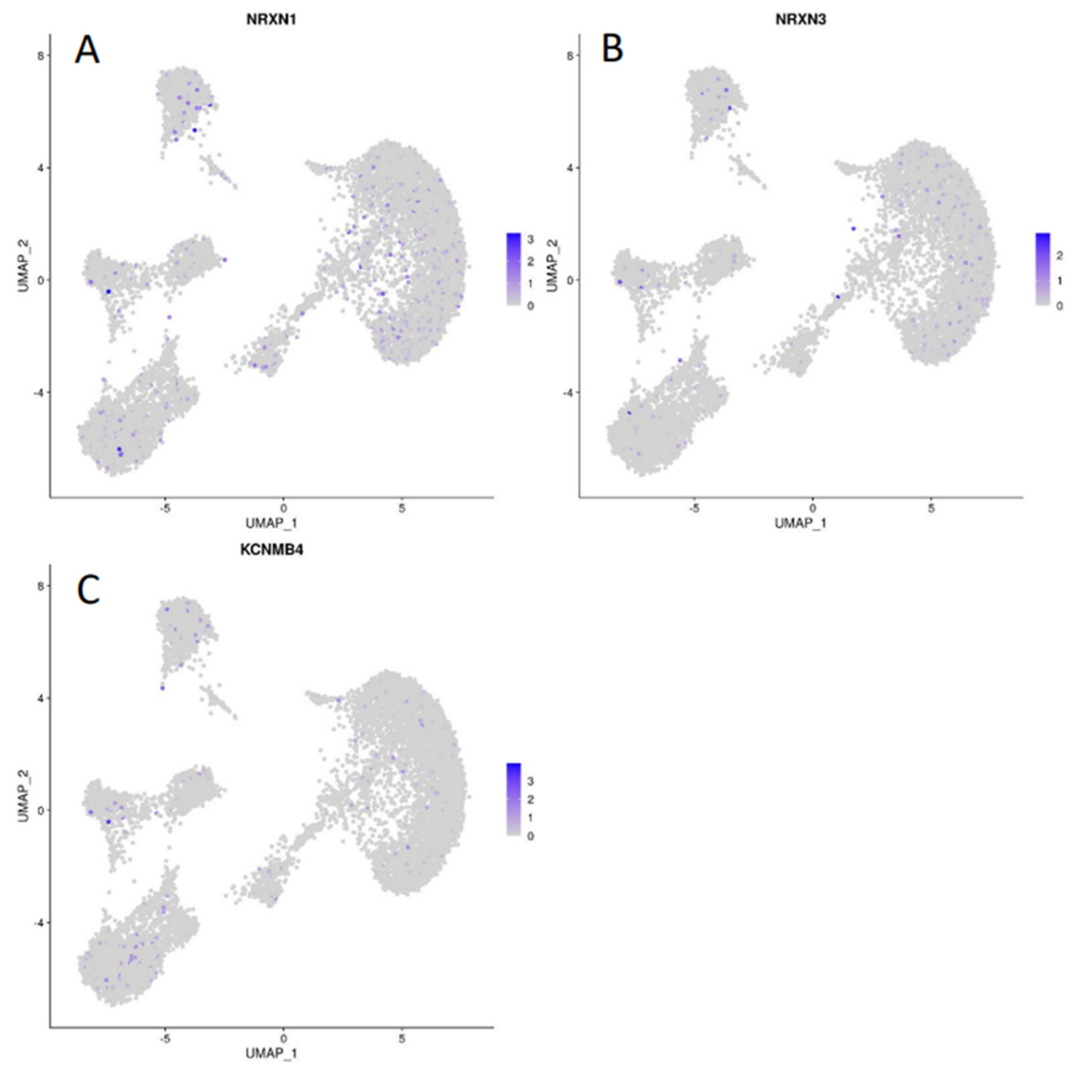

Supplementary Figure S2: Supervised examination of neural cell markers in the cardiac single cell dataset. A) Neurexin 1 (NRXN1). B) Neurexin 3 (NRXN3). C) Potassium Calcium-Activated Channel Subfamily M Regulatory Beta Subunit 4 (KCNMB4)

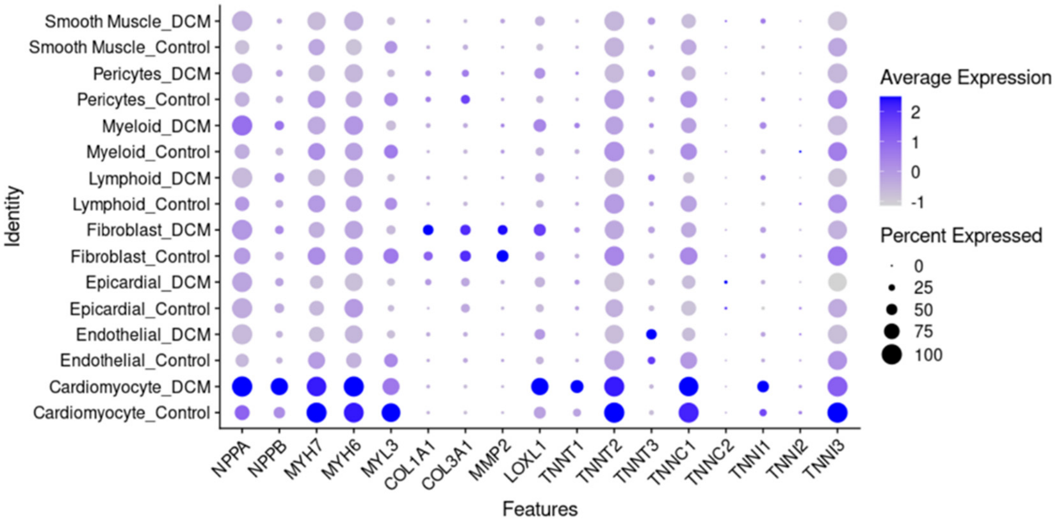

SupplementaryFigureS3: Expression of selected common HF-dysregulated genes across all cardiac cell populations, split into DCM and control (non-HF) cohorts, indicating differences in celltype and cohort expression. Notably NPPA, NPPB, COL1A1 and LOXL1 were increased in DCM, while MYL3 and TNNI3 were decreased.

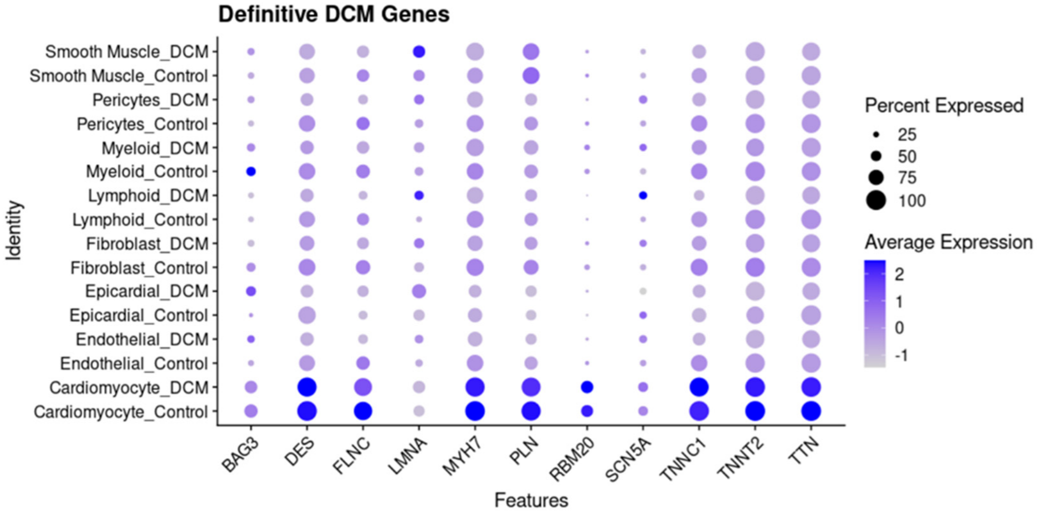

SupplementaryFigureS4: Genes with clear genetic association with DCM as reported by Jordan et al. 2021. Seven (of FLNC, DES, MYH7, PLN, TTN, TNNC1 and TNNT2) of the 11 genes show significant dysregulation in expression in DCM in at least one cardiac celltype.

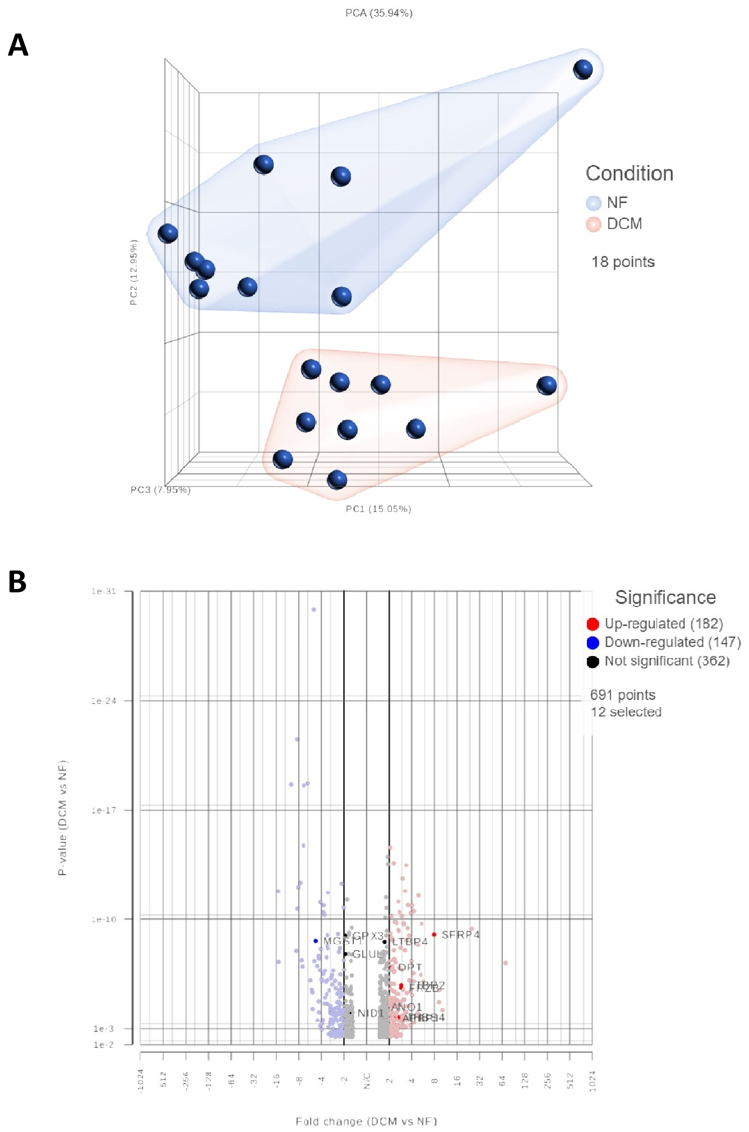

Supplementary Figure S5: Bulk-RNA sequencing analysis of human left ventricular tissue from non-failure patients versus DCM patients. (**A**) Principle components analysis plot depicting difference in principle gene expression components between NF (blue) (n=9) and DCM patients (red) (n=9). (**B**) Volcano plot depicting downregulated genes (blue) and upregulated genes (red) identified in RNA sequencing, selected gene candidates are labelled.

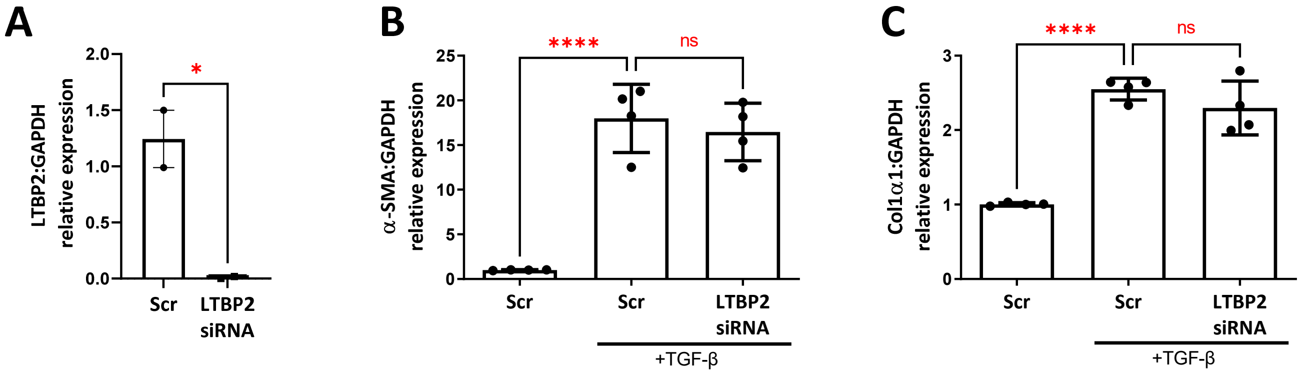

Supplementary Figure S6: The effect of siRNA-targeted knockdown of LTBP2 on the TGFβ response of human cardiac fibroblasts. **A**) RT-PCR analysis of *LTBP2* in HCFs transfected with 25nM *LTBP2* siRNA (or scrambled control) (n=2). RT-PCR analysis of **B**) *αSMA* and **C**) *col1α1* in HCFs transfected with 25nM *LTBP2* siRNA (or scrambled control) in the presence or absence of TGFβ1 (n=4).
